# The macroeconomic burden of noncommunicable diseases in the United States: Estimates and projections

**DOI:** 10.1371/journal.pone.0206702

**Published:** 2018-11-01

**Authors:** Simiao Chen, Michael Kuhn, Klaus Prettner, David E. Bloom

**Affiliations:** 1 Heidelberg Institute of Global Health, Heidelberg University, Heidelberg, Germany; 2 Wittgenstein Centre (IIASA, VID/ÖAW, WU), Vienna Institute of Demography, Vienna, Austria; 3 University of Hohenheim, Institute of Economics, Stuttgart, Germany; 4 Department of Global Health and Population, Harvard T.H. Chan School of Public Health, Boston, Massachusetts, United States of America; Centers for Disease Control and Prevention, UNITED STATES

## Abstract

We develop and calibrate a dynamic production function model to assess how noncommunicable diseases (NCDs) will affect U.S. productive capacity in 2015–2050. In this framework, aggregate output is produced according to a human capital–augmented production function that accounts for the effects of projected disease prevalence. NCDs influence the economy through the following pathways: 1) when working-age individuals die of a disease, aggregate output undergoes a direct loss because physical capital can only partially substitute for the loss of human capital in the production process. 2) If working-age individuals suffer from a disease but do not die from it, then, depending on the condition’s severity, they tend to be less productive, might work less, or might retire earlier. 3) Current NCD interventions such as medical treatments and prevention require substantial resources. Part of these resources could otherwise be used for productive investments in infrastructure, education, or research and development. This implies a loss of savings across the population and hampers economy-wide physical capital accumulation. Our results indicate a total loss of USD94.9 trillion (in constant 2010 USD) due to all NCDs. Mental health conditions and cardiovascular diseases impose the highest burdens, followed by cancer, diabetes, and chronic respiratory diseases. In per capita terms, the economic burden of all NCDs in 2015–2050 is USD265,000. The total NCD burden roughly corresponds to an annual tax rate of 10.8% on aggregate income.

## Introduction

Noncommunicable diseases (NCDs) have decisively replaced infectious diseases and malnutrition as the dominant cause of death globally. They are also the world’s main cause of disability, and their impact is growing over time. Table 1 shows the percentage of total deaths and disability-adjusted life years (DALYs) that NCDs have caused across countries at different stages of sociodemographic development over time according to Global Burden of Disease Study (2016) [1]. NCDs’ rapid growth affects not only developed countries, but also low- and middle-income countries. These trends are reflective of changes in modifiable risk factors (such as smoking, unhealthy diet, lack of physical activity, and the harmful use of alcohol, which lead to overweight and obesity, raised blood pressure, and raised cholesterol) and nonmodifiable risk factors (such as population aging).

**Table 1:** Percentage of total deaths and DALYs caused by NCDs.

Country group	% of total deaths caused by NCDs			% of total DALYs caused by NCDs		
	1990	2000	2016	1990	2000	2016
Global	58	62	72	44	49	61
High SDI^a^ countries	88	89	89	82	84	86
United States	87	88	89	81	83	85
High-middle SDI countries	80	83	87	67	73	79
Middle SDI countries	64	72	80	52	61	73
Low-middle SDI countries	37	42	58	29	34	50
Low SDI countries	25	26	38	19	21	32

^a^ SDI (sociodemographic index) is constructed by Institute for Health Metrics and Evaluation and is a summary measure of a country’s sociodemographic development. It is based on average income per person, educational attainment, and the total fertility rate (TFR). For example, zero SDI represents the lowest income per capita, lowest educational attainment, and highest TFR observed across all Global Burden of Disease geographies from 1980 to 2015, and one represents the highest income per capita, highest educational attainment, and lowest TFR.

In the United States, NCDs account for 89% of all deaths and have long surpassed infectious diseases as the main cause of death [1]. Table 2 shows U.S. prevalence and mortality rates, deaths, and DALYs by specific NCD categories. U.S. prevalence is above average for four of the leading NCDs: cardiovascular diseases, cancer, chronic respiratory diseases, and mental health conditions [1]. Currently, the United States also has a higher mortality rate than the global average for all NCDs and for the five leading NCDs. Across all NCDs, mental and substance use disorders generate substantially higher DALYs in the United States than in other countries. Mental health conditions have a DALY rate of 3,725 per 100,000, which is 1.7 times the global average (i.e., 2,198 per 100,000), or 1.3 times the high-income average (i.e., 2,867 per 100,000) [1].

**Table 2:** Prevalence and mortality rates, deaths, and DALYs by NCD categories in the United States^a^.

NCD categories	Prevalence rate (per 100,000)		Mortality rate (per 100,000)		Total deaths (thousands)		DALYs (millions)	
	1990	2016	1990	2016	1990	2016	1990	2016
**Cardiovascular diseases**	9,321	10,433	357	279	892	901	15	15
**Cancer**	1,586	2,108	206	212	516	685	11	14
**Chronic respiratory diseases**	8,782	9,464	41	59	102	191	3	5
**Diabetes/urog/blood/endo**^b^	35,832	37,385	43	63	108	203	4	7
**Mental and substance use**	19,785	19,793	5	14	13	45	8	12
**All NCDs**	88,227	89,337	749	760	1,858	2,442	60	79

^a^ Source: Global Burden of Disease Study 2016 results [1]

^b^ Diabetes, urogenital, blood, and endocrine diseases

NCDs are noteworthy not only for their prevalence, but also because they may impose a high economic burden that could rise substantially over the coming decades. NCDs’ global economic burden has been estimated at USD47 trillion in 2010–2030 (measured in real USD with the base year 2010)—equivalent to 75% of global gross domestic product (GDP) in 2010 [2, 3]. However, the methodology used to develop this estimate did not account for productivity losses due to morbidity and relied on poor-quality data. This paper aims to construct new estimates for the United States based on a more comprehensive model and better-quality data. In our model, NCDs can affect economic output through several pathways. First, when working-age individuals die of a disease, labor supply is reduced directly. Even if less severe, illness tends to reduce workers’ productivity and labor supply [4–8]. Second, NCD interventions such as medical treatments require substantial resources that could otherwise be used for productive investments in infrastructure, education, training, or research and development (R&D). Using these resources to treat NCDs will impede the accumulation of physical capital, human capital, and R&D, and thereby impinge on macroeconomic performance. Investing in preventing, curing, or slowing the progression of NCDs therefore confers, at least in principle, both health and economic benefits. See Bloom et al. (2018) for a review of economic theory and evidence on the links from health to macroeconomic performance [9].

Apart from its comparatively high disease burden, the U.S. situation is interesting for the following reasons. First, in comparative perspective, the United States has extraordinarily high health expenditures, but does not attain the best health outcomes. In 2015, U.S. per capita health expenditure (current health expenditure in purchasing power parity) was USD9,536, ranking first in the world [10]. However, considering the high expenditure level, the outcomes are disappointing. For example, the U.S. infant mortality rate is 1.24 times that of high-income countries; life expectancy at birth is two years less than the average of high-income countries; the burden of NCDs is higher; and, of the latter, the burden of mental health conditions is substantially higher. Second, the economic impact of NCDs in the United States is likely to be large not only because of high health expenditures and high NCD prevalence, but also because of high productivity losses. With a highly educated population and a per capita GDP of USD57,467, NCDs might have a substantially higher economic impact in the United States than elsewhere. Third, the high quality of data that our model requires is more readily available for the United States, allowing us to generate more accurate projections.

This paper employs a calibrated dynamic macroeconomic model to assess the extent to which NCDs will affect U.S. productive capacity in 2015–2050 and to calculate the resulting economic burden in terms of foregone GDP. Most of the existing literature describes the individual-level effects of health conditions on economic and overall well-being [11]. The works surveyed in [11] address the impact of only one or two diseases, such as chronic obstructive pulmonary disease [12], breast cancer [13], osteoporosis-related fractures [14], or asthma [15], on a specific population group’s economic performance. Productivity losses are often measured in units of labor input (e.g., labor market participation, change in hours worked, medical/sickness leave) rather than output. For example, men with chronic disease worked 6.1% fewer hours and women worked 3.9% fewer hours [16]. Even when using dollars as an outcome measure, most studies focus on the individual as the basis for measurement [17]. For example, among postmenopausal females in an employed population in the United States, the annual direct medical treatment costs were estimated to be USD13,925 for breast cancer, or USD12,055 for cardiovascular diseases, and indirect work-related losses were estimated to be USD8,236 for breast cancer and USD4,990 for cardiovascular diseases [18]. A recent study provides the direct medical spending for 155 conditions in 2013 and finds that diabetes has the highest cost, with an estimated spending of USD101.4 billion, followed by ischemic heart disease, with an estimated spending of USD88.1 billion [19]. Both figures exceed by far the direct costs of prescription opioid overdose, abuse, and dependence (i.e., USD28.9 billion) in the same year [20]. Finally, [21] shows that people with mental health conditions have a significantly higher possibility to use opioids.

None of the aforementioned studies account for economic adjustment mechanisms, such as the substitution of labor lost with other workers or capital. They also do not consider how the loss of labor across age groups with different levels of human capital bears on the total stock of human capital; nor do they consider the impact of treatment costs on the accumulation of physical capital. To estimate the macroeconomic costs of NCDs a rigorous model is required that enables us to describe how labor supply and the capital stock—key determinants of economic growth—are affected by NCDs, while incorporating adjustment mechanisms. Such an approach was pioneered only recently [2, 3, 22, 23]. In this study, we apply the model developed in [23] to calculate the economic burden of all NCDs for the United States and project it over the time span 2015–2050. All NCDs include cardiovascular diseases; cancer; chronic respiratory diseases; cirrhosis; digestive diseases; diabetes; urogenital diseases; blood diseases; endocrine diseases; musculoskeletal disorders; and other noncommunicable diseases, including congenital anomalies, skin and subcutaneous diseases, sense organ diseases, oral disorders, and mental health conditions. We also separate out the results for the five leading NCDs—cardiovascular diseases, cancer, chronic respiratory diseases, diabetes, and mental health conditions.

## Methodology

In the following, we outline why a macroeconomic framework incorporating endogenous adjustment mechanisms is more appropriate than relying on the more common aggregation of individual earnings data. In a competitive economy, the wage is the marginal product of labor (i.e., the change in output that results from employing one additional unit of labor), which can be derived based on the aggregate production function. Owing to a decreasing marginal product of labor, earnings are lower for a given capital stock in a healthy economy without NCDs than in an economy with NCDs. Thus, multiplying the wage level with the number of workers lost tends to overestimate the output loss if one takes the earnings for those observed in the presence of NCDs. The output loss tends to be underestimated if one takes the earnings for the counterfactual case in which NCDs are eliminated. In a dynamic economy, the output loss lies somewhere in between these two estimates. So given that most studies use the earnings for those observed in the presence of NCDs, they tend to overestimate the loss whenever non-marginal variations in labor are present. In dynamic terms, individual-level studies disregard any changes to earnings as driven by productivity growth but more subtly also by changes in physical and human capital stocks. Finally, what earnings-based studies fail to account for is that in the absence of NCDs, the capital stock would be higher, which, in turn, would imply a higher level of earnings. Thus, in that sense they actually tend to underestimate the output loss. Any of these inaccuracies are avoided in our framework, as we directly calculate the output loss associated with the reduction in the supply of labor and capital due to the presence of NCDs.

The underlying macroeconomic approach (the World Health Organization’s EPIC framework, or Projecting the Economic Cost of Ill Health framework) was first developed based on the growth model of Solow [24] and applied to estimate NCDs’ worldwide economic burden [25]. Since its development, researchers have applied the EPIC framework to several countries including China and India [3, 22]. The advantage of EPIC is that it allows for economic adjustment mechanisms and accounts for physical capital and labor loss from mortality. Later the model was improved [26] and applied to Latin American countries, including Costa Rica, Jamaica, and Peru [27]. The framework in this study is based on a human capital–augmented production function, as in [28], that accounts for the effects of projected disease prevalence on physical and human capital. Unlike EPIC, which captures mortality effects only, our model also accounts for NCDs’ effects on aggregate output by reducing effective labor supply through morbidity and by reducing physical capital accumulation through higher health expenditures or treatment costs. Our framework also contributes by reflecting aggregate macroeconomic effects when the impact of NCDs varies by age and when age groups differ in (average) education and experience—dimensions of heterogeneity that so far have not been considered. Thus, previous studies may mis-estimate NCDs’ overall economic burden because they do not fully consider all the pathways. Our framework was first applied to East Asian countries, including China, Japan, and South Korea [23].

In applying the model, we first recognize that NCDs can affect the economy via effective labor supply due to mortality and morbidity. Disease-induced mortality reduces the population and hence the number of working-age individuals. At the same time, disease related impairments, chronic pain, and mental health conditions tend to reduce worker productivity, increase absenteeism, and induce earlier retirement. Indeed, for some diseases, such as mental health conditions, the morbidity effect exerts a stronger economic impact than the mortality effect. We also consider the age-specific average human capital of the labor force in this analysis because NCDs may disproportionately affect more experienced age groups. Finally, physical capital accumulation is reduced because public and private resources are diverted to finance treatment costs. The health-insured part of treatment costs, in turn, translates into higher private health insurance premiums and Medicare/Medicaid taxes. This reduces aggregate savings and hampers economy-wide physical capital accumulation.

### Data sources

The GDP projections for the status quo scenario and the saving rate are taken from the World Bank’s database [29–31]. The mortality and morbidity data (i.e., years of life lost due to premature mortality (YLL) and the years lost due to disability (YLD)) are from the Global Burden of Disease Study [1]. We rely on the International Labour Organization for age-specific labor force projections [32], the Barro-Lee education database for age-specific data on average years of schooling [33], and a World Bank report for returns to schooling data [34]. We calculate human capital according to the Mincer equation [35] with parameters from [36]. The physical capital data and the capital income share are from the Penn World Tables [37, 38]. The S1 Appendix describes the parameter values and data sources used in the macroeconomic model in detail.

We obtain information on treatment costs and the disease-specific annual growth rates of medical expenditures from Dieleman et al. (2016) [19]. In this study, the spending estimates were adjusted to account for comorbidities. In our projections, we assume a fixed annual growth rate of the disease-specific per capita treatment costs to adjust for rising medical costs over time. The annual growth rate of the per capita treatment costs is calculated based on the average annual growth rate of the total treatment costs over 1996 to 2013 in [19], cleaned of population growth. Table 3 displays the estimates of the treatment costs per capita and their growth rates based on [19].

**Table 3:** Treatment cost in the United States by aggregated condition category.

Disease category	National total expenditure in 2013 (billions of 2010 USD)^a^	Annualized total rate of change, 1996–2013, %^a^	Treatment costs per capita in 2015 (in 2010 USD) ^b^	Annualized per capita rate of change, %^c^
Cardiovascular diseases	212.6	1.2	719.0	0.24
Cancer	106.2	2.5	359.2	1.52
Chronic respiratory diseases	121.6	3.7	411.2	2.71
Diabetes	93.3	6.1	315.5	5.09
Mental health conditions	172.8	3.7	584.4	2.71
All NCDs (including other NCDs)^d^	1353.3	3.4	4,576.7	2.38

^a^ Data source: Dieleman et al. (2016) [19].

^b^ The 2015 estimates account for health expenditure growth between 2013 and 2015.

^c^ The annualized per capita rate of change is adjusted for population growth from 1996–2013.

^d^ In addition to the five leading NCDs that are singled out in the table, all NCDs include cirrhosis; digestive diseases; diabetes; urogenital diseases; blood diseases; endocrine diseases; musculoskeletal disorders; and other noncommunicable diseases, including congenital anomalies, skin and subcutaneous diseases, sense organ diseases, and oral disorders.

### Scenarios and model

To quantify the economic burden of a particular disease category we compare economic performance, as measured by cumulative GDP over the period 2015–2050, between the following two scenarios: 1) The status quo scenario, in which no interventions are implemented that would reduce mortality from NCDs further than projected, and 2) a counterfactual scenario in which we assume the complete (or partial) elimination at zero cost of classes of NCDs or of all NCDs from 2015–2050. We then calculate the macroeconomic burden of a particular class of NCDs (or of all NCDs) as the undiscounted cumulative difference in projected annual GDP between these two scenarios.

Using our model–described in detail in [23] and in the S1 Appendix–we construct economic projections for each scenario, which allows us to quantify the impact of NCDs on aggregate output. The model is based on the production function of Lucas’s [28] human capital-based economic growth model, which allows us to consider the aggregate macroeconomic effects when the impact of NCDs varies by age and when age groups differ in (average) education and experience. The baseline model assumes that aggregate income growth depends on technological progress, physical capital accumulation, and human capital accumulation. For more details, see the S1 Appendix.

## Main results

**Fig 1:**
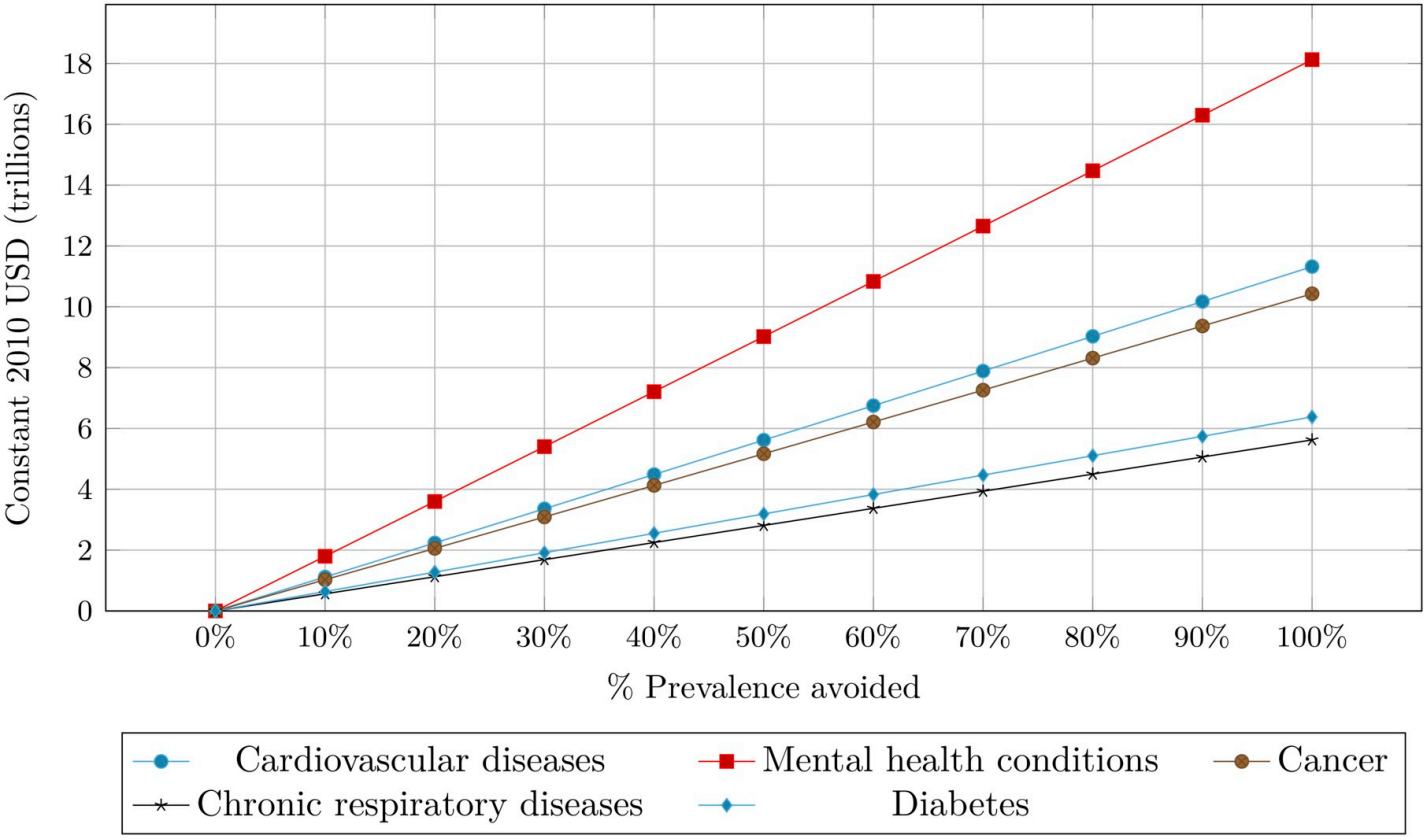
Estimates of GDP that could be gained per percent reduction in disease prevalence in the United States, 2015–2050 (in trillions of 2010 USD).

The following presents direct estimates of the macroeconomic burden of the five leading NCDs—cardiovascular diseases, cancer, chronic respiratory diseases, diabetes, and mental health conditions—and of all NCDs in the United States. Fig 1 shows by disease how much output could be gained per percentage reduction in disease prevalence. For example, the 10% estimate compares the cumulative difference in U.S. GDP between the status quo scenario with disease prevalence as predicted and the counterfactual scenario, in which disease prevalence is reduced by 10%. The results are displayed in trillions of constant 2010 USD and are cumulative over the period 2015–2050. Our estimates indicate that the costliest conditions in the United States are mental health conditions and cardiovascular diseases, followed by cancer and diabetes. Chronic respiratory diseases generate the lowest burden. Cardiovascular diseases and mental health conditions have the highest treatment costs among the five disease categories, which partially explains these results. Furthermore, compared with other NCDs, mental health conditions have high morbidity rates in the working-age population, which explains why they generate such a high economic burden.

Table 4 shows the estimates of NCDs’ total economic impact in the five leading categories and for all NCDs in the United States. The results indicate that the total economic cost of chronic conditions in 2015–2050 is USD94.9 trillion.

**Table 4:** Estimates of lost GDP due to five leading NCDs and due to all NCDs in the United States, 2015–2050 (in trillions of 2010 USD).

Disease	Total disease burden (trillions of 2010 USD)
Cardiovascular diseases	11.3
Cancer	10.4
Chronic respiratory diseases	5.6
Diabetes	6.4
Mental health conditions	18.1
All NCDs (including other NCDs)^a^	94.9

^a^ See footnote in Table 3.

We also investigate the contribution of treatment costs to our estimates of the total disease burden. Table 5 shows the model estimates with the treatment costs excluded. In the United States, treatment costs account for 39% of NCDs’ total economic burden. We calculate the absolute value of the treatment cost effect as the output difference between the scenario in which we consider all effects and a scenario in which we only consider the mortality and morbidity impact but not the impact of treatment costs. Comparing across diseases shows that the effect of treatment costs is highest for diabetes and chronic respiratory diseases, followed by cardiovascular diseases. Recall that the contribution of treatment costs to the disease burden depends on the fraction of the treatment cost that is diverted from savings and, therefore, decreases capital accumulation. In our benchmark projection, we assume this fraction to equal the saving rate. We also conduct a sensitivity analysis and find that the treatment cost effect (39% for all NCDs) ranges from 37% to 41% if we increase or decrease the share of treatment costs financed from savings by 10%. See the S1 Appendix for more details.

**Table 5:** Estimates of foregone GDP due to the five leading NCDs and due to all NCDs excluding the treatment cost effect in the United States, 2015–2050 (trillions of 2010 USD).

Disease category	Total disease burden excluding the impact of treatment cost (trillions of 2010 USD)^a^	Treatment cost effect^b^
Cardiovascular diseases	7.0	38%
Cancer	7.8	25%
Chronic respiratory diseases	2.1	63%
Diabetes	2.4	63%
Mental health conditions	13.1	28%
All NCDs (Including other NCDs)^c^	57.6	39%

^a^ i.e. we only consider the effect of mortality and morbidity

^b^ Treatment cost effect = the percentage of NCDs’ total economic burden attributable to treatment costs

^c^ See footnote in Table 3.

To put the numbers in perspective, Table 6 provides alternative yardsticks of the total disease burden (100% of prevalence averted) due to all NCDs. The total disease burden is 569% of aggregate GDP in 2015. The per capita figures are based on the average population size in 2015–2050 and amount to a loss of USD265,000 per person (in constant 2010 USD). The third measure in Table 6 shows the total U.S. NCD burden related to the total output for the period 2015–2050 adjusted for the projected growth rate. According to this representation, the burden of NCDs is equivalent to an annual tax of 10.8% on aggregate income in the United States. While this number might seem high at first glance, the total health expenditures in the United States amount to 17% of GDP and this figure does not include any lost output because of mortality or morbidity. [39]

**Table 6:** Disease burden (100% of prevalence averted) due to all NCDs in the United States in 2015–2050 for different measures of economic performance.

	Total disease burden (trillions of 2010 USD)	% of 2015 GDP	Per capita loss (2010 USD)	% of total GDP in 2015–2050
**Estimates for 100% reduction of disease burden**	94.9	569%	265,000	10.8%

## Discussion

### The NCD burden in the United States

The projected macroeconomic burden for all NCDs in 2015–2050 in the United States is large at the aggregate and per capita levels, as shown in Table 6. For comparison, we calculated tax rates of 3.4%, 2.8%, and 3.4%, respectively, for China, Japan, and South Korea (and the time frame 2015–2030) [23]. A notable comparison is the 39% contribution that the treatment cost effect (= total economic burden of treatment cost–induced savings reductions) makes to the total economic burden of NCDs that we find for the United States against 56%, 22%, and 38% in China, Japan, and South Korea. While the comparatively high contribution of treatment costs to China’s economic burden of NCDs is initially surprising, our previous study [23] shows that this results from China’s high gross savings rate of close to 50%. Given our assumption that treatment costs are paid out of savings in direct proportion to the saving rate, a country with a larger saving rate will experience a stronger effect of NCDs on capital accumulation. In the United States, the gross saving rate is only 19% and thus lower than Japan’s at 22% and South Korea’s at 34%. Still, in the United States, treatment costs contribute a higher percentage share to its economic burden of disease than in Japan and South Korea. One reason is that the United States has an exceptionally high share of health expenditures in terms of GDP, which in 2015 was as high as 17% compared with around 10% globally, 11% in Japan, 7.4% in South Korea and 5.3% in China [39, 40].

### The high economic burden of mental health conditions

Although cardiovascular diseases and cancer are the two leading NCDs in terms of U.S. mortality, they have less of an effect on economic growth than mental health conditions do. The reasons are as follows. First, mental health conditions are more likely to affect working-age individuals. Although cardiovascular diseases and cancer have higher mortality rates, most of the mortality affects cohorts aged 65 and older who are retired or do not contribute to the formal economy as much as younger cohorts with higher employment rates. For example, in 2016 mortality rates in the 30–35 age group for mental health conditions were 1.25 times those for cardiovascular diseases for women and 1.67 times for men, respectively [1]. A similar argument holds true in the case of cancer.

Second, even if mental health conditions do not lead to death, they come with much higher morbidity than cardiovascular diseases and cancer. For example, the YLDs for all ages in 2015 were 1,162 for cancer and 1,722 for cardiovascular diseases, but 9,624 for mental health conditions [41]. An individual who lives and works with depression, for example, will tend to experience much lower labor supply and productivity [42].

Third, both treatment cost and its growth rate for mental health conditions are very high. In 2015, total expenditures on mental health conditions in the U.S. amounted to USD173 billion, or USD584 per capita (in constant 2010 USD) and thus to more than 160% of the cost of treating cancer, which is USD106 billion or USD359 per capita. The annual growth rate of treatment costs for mental health conditions is 3.7%, as compared to 1.2% for cardiovascular disease and 2.5% for cancer.

### Implications for health policy

Insofar as our study shows that NCDs impose a substantial economic burden, it adds to the economic justification for tackling NCDs. Unlike most previous studies, which estimate only one or several NCDs or risk factors in one year, we estimate and project the economic burden of all NCDs from 2015 to 2050 for the U.S. The results presented indicate a substantial benefit from promoting NCD prevention strategies (e.g., focusing on behavioral risk factors such as obesity, smoking, and the unsafe use of alcohol). Nearly 40% of the U.S. population is obese, and reducing this number by promoting healthy diet and physical activity would be beneficial.

In addition, because NCD-related treatment costs contribute to nearly 40% of the economic burden of NCDs, substantial benefits will be enjoyed by lowering those treatment costs. Medical expenditure is higher and has grown more rapidly in the United States than in other countries with better health outcomes [43, 44]. High prices are one important driver of health expenditures [45]. The average price of a bypass surgery, for instance, was the highest in the United States—almost twice the price of that in the second-most expensive country, Australia [44]. It is estimated that about one-third of medical spending in the United States is wasted due to excessive prices, unnecessary services, failures of care delivery and care coordination, administrative costs, and fraud and abuse [46]. Recent research also indicates that some highly cost-effective services are underused (e.g. beta blockers, telemedicine) [47], while some less cost-effective services are overused (e.g., coronary angiography, carotid endarterectomy, and coronary artery bypass grafting) [48, 49].

Our results reveal that mental health conditions impose the highest economic burden. Thus paying more attention to them may be cost-beneficial for the government [50]. Currently, the overuse of opioids is an emerging public health threat in the United States, and evidence shows that adults with mental health disorders are significantly more likely to use opioids [21].

Our findings support the World Health Organization’s call for universal health coverage [51]. Currently, the United States has lower healthcare access than other wealthy nations [41, 52]. The introduction of the Affordable Care Act has increased coverage, with the share of the uninsured population falling from 14% in 2013 to 9% in 2015. Still, insurance covers only 91% of the population, which is the second lowest coverage rate after Greece among all Organisation for Economic Co-operation and Development (OECD) countries. This is problematic given that 22% of the population skipped consultations because of high costs in 2016, which is more than twice the OECD average (i.e., 9.1%) [43]. Conversely, evidence exists that universal health coverage improves health [41]. Thus, the absence of universal health care may actually raise the costs of the healthcare system in the long run.

### Strengths of our model and potential improvements

Our framework accounts for the impact of NCDs on economic growth through mortality, morbidity, and treatment cost effects. By considering a macroeconomic production function based on labor, human capital, and physical capital, our model conveniently allows us to abstract from complex inter-sectoral adjustment processes, while at the same time it internalizes the equilibrium effects of changing labor and capital supplies which are crucially important for assessing the large-scale dynamic impacts associated with the elimination of NCDs. We also incorporate age-specific human capital to account for education-related productivity differences among members of different cohorts who are differentially affected by NCDs. See the S1 Appendix for more modeling details.

Because many people have more than one NCD, we use the treatment costs, YLL, and YLD data that adjust for the effects of comorbidities [19, 53]. In the treatment cost estimation, rather than attributing all of the treatment costs to the primary diagnosis, the adjusted spending estimate is attributed to each condition, by incorporating the information on multiple secondary diagnoses and using a previously developed regression-based method [19]. A multiplicative model is used to estimate the health burden adjusted for comorbidity [53]. We may underestimate the economic benefit from partially eliminating NCDs or from eliminating certain groups of NCDs. For example, the value of eliminating diabetes not only saves the treatment costs of diabetes, but also some portion of the treatment costs of cardiovascular diseases, since people with diabetes are likely to get cardiovascular diseases as well. Comorbidities also have implications for our assumption that life expectancy equals average life expectancy if a disease is averted. Assume, for example, a person has cancer but dies of a heart attack before dying of cancer. Then eradicating cardiovascular diseases would not imply that this person has the population’s average life expectancy, but that he or she would only have the average life expectancy of other individuals with cancer. Incorporating this into our projections is difficult, however, because it would require following the distinct disease histories of individuals or small clusters of individuals.

While our framework allows for a uniquely comprehensive analysis of NCDs’ macroeconomic burden, it does not yet allow for interaction effects between diseases and individual choices in regard to savings [54–56], human capital [57, 58], and labor supply [59, 60]; nor does it allow for public and private choices regarding the supply of health care or R&D expenditures. Recent theoretical contributions have studied such behavioral channels [61, 62], but including them into the current macroeconomic framework is beyond the scope of our analysis. However, it is worthwhile for future research to extend the model so that treatment costs can affect human capital accumulation as well. In addition, sustaining the NCD elimination status could result in health maintenance costs. If these costs are accounted for, the (relative) economic costs of NCDs will be lower. A final limitation of our model is that we do not consider the correlation between education and mortality due to lack of data.

## Supporting information

S1 Appendix Model, data, and sensitivity analysis. (DOCX)
