# Supplementary material for: The macroeconomic burden of noncommunicable diseases in the United States: Estimates and projections
Source: PLoS One. 2018 Nov 1;13(11):e0206702. doi: 10.1371/journal.pone.0206702 (PMC6211719; doi:10.1371/journal.pone.0206702)
Supplement: S1 Appendix — (DOCX) [file pone.0206702.s001.docx]

**S1 Appendix. Model, data, and sensitivity analysis**

In this S1 Appendix to the PLOS ONE paper “The macroeconomic burden of noncommunicable diseases in the United States: Estimates and projections,” we provide additional details related to our study, including the mathematical formulation of our model, information on the data sources, and a sensitivity analysis.

**A. Mathematical formulation**

**Modeling details**

We aim to quantify the impact of noncommunicable diseases (NCDs) on economic output in the United States. For each disease, we first identify the disease burden (in terms of mortality, morbidity, and treatment cost). Then we construct projections for the U.S. economy in the two scenarios of interest— the status quo, without any changes to projected disease prevalence, and the counterfactual scenario with a predefined reduction in disease prevalence—following the model in [1]. We calculate the total economic loss for the United States as the undiscounted cumulative difference in projected annual gross domestic product (GDP) between the status quo scenario and the counterfactual scenario with a 100% reduction in disease prevalence. The following detailed model description follows our previous contribution [1], in which we applied the framework to China, Japan, and South Korea.

**Production function**

Consider an economy in which time $t\in[0,\infty)$ evolves discretely. Building upon Lucas [2], we consider the following production function for the U.S. economy:

$$\begin{aligned} Y_{t}=A_{t}K_{t}^{\alpha}H_{t}^{1-\alpha} ,\#\left( 1 \right) \end{aligned}$$

where $Y_{t}$ is aggregate output; $A_{t}$ is the technological level at time $t$, which we assume to evolve exogenously; $K_{t}$ is the physical capital stock (i.e., machines, factory buildings, etc.); and $H_{t}$ represents aggregate human capital. The parameter $\alpha$ is the elasticity of final output with respect to physical capital. The aggregate production function recognizes that output is not only produced with physical capital and *raw labor* as in the Solow framework [3], on which the original EPIC model is based [4], but with *effective labor,* of which health is a crucial determinant.

Physical capital evolves according to

$$\begin{aligned} K_{t+1}=\left( 1-\delta\right)K_{t}+Y_{t}-C_{t}-\sum_{j\in I} TC_{j,t}=\left( 1-\delta\right)K_{t}+s_{t}Y_{t} ,\#\left( 2 \right) \end{aligned}$$

where $\delta$ is the rate of depreciation, $C_{t}$ is consumption, $s_{t}$ is the saving rate, and $TC_{j,t}$ is the treatment cost for disease $j\in I$, where $I$ is the set of diseases that we consider. From Equation (2), it follows that the saving rate is defined as

$s_{t}=1-\frac{C_{t}+\sum_{j\in I} TC_{j,t}}{Y_{t}}$.

Note that aggregate output $Y_{t}$ is used for three purposes: 1) to pay total treatment costs $\sum_{j\in I} TC_{j,t}$; 2) to consume the amount $C_{t}$; and 3) to save. Note that $TC_{j,t}$ refers to disease-specific costs of ongoing treatment and other intervention efforts. In general, these costs are best characterized by the direct costs of health care, including hospitalization, medication, etc.

Individuals of age group $a$ are endowed with $h_{t}^{(a)}$ units of human capital and supply $\mathcal{l}_{t}^{(a)}$ units of labor from age 15 up to their retirement at age $R$, i.e., for $a\in[15, R]$. Children below the age of 15 and retirees above the age of $R$ do not work. In our analysis, we use labor projections by the International Labour Organization. Positive entries for the labor force exist for cohorts above the age of 65. Aggregate human capital in the production function (1) is then defined as the sum over the age-specific effective labor supply of each age group:

$$\begin{aligned} H_{t}=\sum_{a=15}^{R} h_{t}^{\left( a \right)}\mathcal{l}_{t}^{\left( a \right)}N_{t}^{\left( a \right)} ,\#\left( 3 \right) \end{aligned}$$

where $N_{t}^{a}$ denotes the number of individuals in age group $a$. Note that aggregate human capital increases with the number of working-age individuals who live in the economy (i.e., with a higher $N_{t}=\sum_{a=15}^{R} N_{t}^{(a)}$), with individual human capital endowment (i.e., with a higher $h_{t}^{(a)}$ for at least one $a$), and with labor supply (i.e., with a higher $\mathcal{l}_{t}^{(a)}$ for at least one $a$).

We follow Mincer [5] and construct average human capital of the cohort aged $a$ according to an exponential function of education and work experience:

$$\begin{aligned} h_{t}^{\left( a \right)}=\exp\left[ \eta_{1}\left( ys_{t}^{\left( a \right)} \right)+\eta_{2}\left( a-ys_{t}^{\left( a \right)}-5 \right)-\eta_{3}\left( a-ys_{t}^{\left( a \right)}-5 \right)^{2} \right],\#\left( 4 \right) \end{aligned}$$

where $\eta_{1}$ is the semi-elasticity of human capital with respect to average years of education as given by $ys_{t}^{\left( a \right)}$, and $\eta_{2}$ and $\eta_{3}$ are the semi-elasticities of human capital with respect to the experience of the workforce $\left( a-ys_{t}^{\left( a \right)}-5 \right)$ and the experience of the workforce squared $\left( a-ys_{t}^{\left( a \right)}-5 \right)^{2}$, respectively.

**Impact of NCDs**

The evolution of labor supply in the status quo scenario is given by

$$\begin{aligned} L_{t}^{\left( a \right)}=\mathcal{l}_{t}^{\left( a \right)}N_{t}^{\left( a \right)}\mathrm{with} N_{t}^{\left( a \right)}=\left[ 1-\sigma_{t-1}^{\left( a-1 \right)} \right]N_{t-1}^{\left( a-1 \right)},\#\left( 5 \right) \end{aligned}$$

where $\sigma_{t}^{\left( a \right)}$ is the overall mortality rate of age group $a$. NCD mortality and morbidity reduce effective labor supply. The mortality effect is captured in the reduction of the population size $N_{t}^{\left( a \right)}$.

Denote the mortality rate of people in age group $a$ due to the disease $i$ by $\sigma_{i,t}^{\left( a \right)}$ and let $\sigma_{-i,t}^{\left( a \right)}$ be the overall mortality rate due to causes other than disease $i$. Then we have

$\left( 1-\sigma_{t}^{\left( a \right)} \right)=(1-\sigma_{i,t}^{\left( a \right)})(1-\sigma_{-i,t}^{\left( a \right)})$.

Now we consider the mortality effect of disease $i$. In general, it reduces labor supply by reducing the population $N_{t}^{\left( a \right)}$ (through $\sigma_{i,t}^{\left( a \right)}$). In the counterfactual case, where the disease is eliminated from time $t=0$ onward, the evolution of labor supply is defined similarly to Equation (5), but with a different overall mortality rate ($\sigma_{-i,t}^{\left( a \right)}$ instead of $\sigma_{t}^{\left( a \right)}$). For simplicity, we assume that the number of births is the same in both cases at each point in time $t$. In general, this is a good approximation because most NCDs affect older adults who contribute little to overall fertility.

In the counterfactual scenario, the size of the cohort aged $a$ at time $t (\bar{N}_{t}^{(a)})$ evolves according to

$\bar{N}_{t}^{(a)}=\left[ 1-\sigma_{-i,t-1}^{\left( a-1 \right)} \right]\bar{N}_{t-1}^{(a-1)}, \bar{N}_{0}^{(a)}=N_{0}^{\left( a \right)}, \bar{N}_{t}^{(0)}=N_{t}^{\left( 0 \right)}$.

Following [1], the loss of labor due to mortality accumulates over the years according to

$\bar{N}_{t}^{(a)}=N_{t}^{\left( a \right)}/\prod_{\tau=0}^{\min\left\{ t,a \right\}-1} \left[ 1-\sigma_{i,t-1-\tau}^{\left( a-1-\tau\right)} \right]$.

The morbidity effect is captured in the reduction of the labor participation rate $\mathcal{l}_{t}^{\left( a \right)}$ because people with an illness typically reduce their labor supply either by reducing working hours or by leaving the work force. Following [1], the labor participation rate in the counterfactual scenario ${\bar{\mathcal{l}}}_{t}^{(a)}$ can be calculated as:

${\bar{\mathcal{l}}}_{t}^{(a)}\approx\mathcal{l}_{t}^{\left( a \right)}/\prod_{\tau=0}^{\min\left\{ t,a \right\}-1} \left[ 1-p_{i}^{\tau}\sigma_{i,t-1-\tau}^{\left( a-1-\tau\right)}\xi_{i}^{\left( a-1-\tau\right)} \right]$,

where $\xi_{i}^{\left( a \right)}$ measures the size of the morbidity effect relative to the relevant mortality rate, and where $p_{i}^{t}$ is the probability that a sick person fails to recover from the disease until time $t$.

Because the impact of morbidity is hard to estimate directly, we first define

$$\begin{aligned} \xi_{i}^{\left( a \right)}=\frac{loss of labor due to morbidity in age group a}{loss of labor due to mortality in age group a} .\#\left( 6 \right) \end{aligned}$$

Next, we assume that the following holds in any given year for each age group $a$:

$$\begin{aligned} \xi_{i}^{\left( a \right)}=\frac{YLD_{i}^{\left( a \right)}}{YLL_{i}^{\left( a \right)}} ,\#\left( 7 \right) \end{aligned}$$

where $YLD_{i}^{\left( a \right)}$ represents the years lived with disease $i$ and $YLL_{i}^{\left( a \right)}$ represents the years of life lost due to disease $i$. Notice that $\xi_{i}^{\left( a \right)}$ can be calculated from the corresponding disability-adjusted life years data reported by the Global Burden of Disease Study [6].

In sum, by reducing the prevalence of NCDs, the *counterfactual scenario* is associated with an increase in labor supply as compared with the *status quo scenario.* We approximate the change in labor supply (at time $t$ for age group $a$) by

$$\begin{aligned} \Delta L_{t}^{\left( a \right)}\approx\mathcal{l}_{t}^{\left( a \right)}N_{t}^{\left( a \right)}\sum_{\tau=0}^{\min\left\{ t,a \right\}-1} \sigma_{i,t-1-\tau}^{\left( a-1-\tau\right)}\left[ 1+p_{i}^{\tau}\xi_{i}^{\left( a-1-\tau\right)} \right] .\#\left( 8 \right) \end{aligned}$$

For the more general case of a partial reduction in the disease prevalence by a factor $\rho$, we obtain the loss of labor for age group $a$ at time $t$ as

$\Delta L_{t}^{\left( a \right)}(\rho)\approx\mathcal{l}_{t}^{\left( a \right)}N_{t}^{\left( a \right)}\sum_{\tau=0}^{\min\left\{ t,a \right\}-1} \rho\sigma_{i,t-1-\tau}^{\left( a-1-\tau\right)}\left[ 1+p_{i}^{\tau}\xi_{i}^{\left( a-1-\tau\right)} \right]$.

The detailed mathematical proof can be found in [1]

NCDs also impede physical capital accumulation in that savings are used to finance a part of the treatment costs. Following [1], physical capital accumulation in the counterfactual scenario can be written as

$$\begin{aligned} \bar{K}_{t+1}=\bar{s}_{t}\bar{Y}_{t}+\left( 1-\delta\right)\bar{K}_{t} ,\#\left( 9 \right) \end{aligned}$$

$\bar{s}_{t}\bar{Y}_{t}=\bar{I}_{t}=\bar{Y}_{t}-\bar{C}_{t}-\sum_{j\in I,j\neq i} TC_{j,t}=s_{t}\bar{Y}_{t}+\chi TC_{i,t}$,

where an overbar indicates that the corresponding variable refers to the counterfactual scenario. In the second expression, $\bar{s}_{t}$ is the saving rate in the counterfactual scenario, where a particular disease $i\in I$ is averted at time $t$. This saving rate is defined by

$$\bar{s}_{t}=\frac{s_{t}\bar{Y}_{t}+\chi TC_{i,t}}{\bar{Y}_{t}} ,$$

where $\chi$ is the fraction of the treatment cost $TC_{i,t}$ that is diverted to savings.

Because disease $i$ is assumed to be eliminated in the counterfactual scenario, the resources that were devoted to its treatment can now be used for saving or for consumption. Notice that this creates an income effect that, in reality, could affect the division of households’ income between saving and consumption. For tractability, we assume that aggregate investment consists of two parts in the counterfactual scenario: a fixed share $s_{t}$ of total output and an additional part from $TC_{i,t}$ that would otherwise have been used to pay to treat disease $i$:

$\bar{I}_{t}=s_{t}\bar{Y}_{t}+\chi TC_{i,t}$.

Similarly, for the case of a partial reduction in disease prevalence by $\rho$, we have

$\bar{I}_{t}=s_{t}\bar{Y}_{t}+\rho\chi TC_{i,t}$.

The intuition is that if the disease is partially eliminated, the treatment cost that is diverted to savings should be added back proportionally.

**B. Data sources**

Table S1 reports the parameter values we employ for our numerical analysis.

**Table S1. Parameter values and data sources**

| Parameter | Value | Source |
| --- | --- | --- |
| $\alpha$ | 0.40 | [7] |
| $\delta$ | 0.05 | [8-10] |
| *S* | 19.2%^a^ | [11] |
| $\eta_{1}$ | 0.133 | [12] |
| $\eta_{2}$ | 0.1301 | [13] |
| $\eta_{3}$ | −0.0023 | [13] |
| $\xi_{i}$ | Depends on disease | [6] |
| $\chi_{i}$ | Set as savings rate | [11] |

^a^ This is the 2016 saving rate for the United States. We assume that the saving rate stays constant throughout the projection period.

**C. Sensitivity analysis**

The contribution of treatment costs to the disease burden depends on the choice of χ (the fraction of the treatment cost that is diverted to savings). In the paper, χ equals the saving rate. In this section, we conduct a sensitivity analysis for χ being greater or smaller than the saving rate.

Table S2 presents the results of reducing or increasing the treatment cost effect χ by 10%.

**Table S2. Estimates of treatment cost effect is** χ **is reduced or increased by 10%**

| Disease | Treatment cost effect if χ is reduced by 10% | Treatment cost effect if χ is increased by 10% |
| --- | --- | --- |
| Cardiovascular diseases | 36% | 40% |
| Cancer | 23% | 27% |
| Chronic respiratory diseases | 60% | 65% |
| Diabetes | 60% | 64% |
| Mental health conditions | 26% | 30% |
| All NCDs (Including other NCDs) | 37% | 41% |

**References**

1. Bloom DE, Chen S, Kuhn M, McGovern ME, Oxley L, Prettner K. The economic burden of chronic diseases: estimates and projections for China, Japan, and South Korea. The Journal of the Economics of Ageing. 2018;forthcoming.

2. Lucas RE. On the mechanics of economic development. Econometric Society Monographs. 1998 Nov 13;29:61–70.

3. Solow RM. A contribution to the theory of economic growth. The Quarterly Journal of Economics. 1956;70(1):65–94.

4. Abegunde D, Stanciole A. An estimation of the economic impact of chronic noncommunicable diseases in selected countries. Geneva, Switzerland: World Health Organization, Department of Chronic Diseases and Health Promotion; : 2006.

5. Mincer J. Schooling, experience, and earnings. Human Behavior & Social Institutions No. 2. 261 Madison Ave., New York, New York 10016: National Bureau of Economic Research Inc.; 1974.

6. Institute for Health Metrics and Evaluation. Global burden of disease study 2016 (GBD 2016) Results. Seattle, United States: Institute for Health Metrics and Evaluation, 2016.

7. University of Groningen and University of California. Share of Labour Compensation in GDP at Current National Prices for United States [LABSHPUSA156NRUG], retrieved from FRED, Federal Reserve Bank of St. Louis Davis 2018 [cited 2018 Aug 23]. Available from: <https://fred.stlouisfed.org/series/LABSHPUSA156NRUG>.

8. US Department of Commerce BoEA. Fixed Assets and Consumer Durable Goods in the United States, 1925–97. US Government Printing Office Washington, DC; 2003.

9. Fraumeni B. The measurement of depreciation in the US national income and product accounts. SURVEY OF CURRENT BUSINESS-UNITED STATES DEPARTMENT OF COMMERCE. 1997;77:7-23.

10. Prettner K. A note on the implications of automation for economic growth and the labor share. Macroecon Dynam. 2017:1-8.

11. World Bank. World Bank database, gross savings (% of GDP) 2018 [cited 2018 March 20]. Available from: <https://data.worldbank.org/indicator/NY.GNS.ICTR.ZS>.

12. Montenegro CE, Patrinos HA. Comparable estimates of returns to schooling around the world. Washington, DC: World Bank Group, 2014.

13. Heckman JJ, Lochner LJ, Todd PE. Earnings functions, rates of return and treatment effects: The Mincer equation and beyond. Handbook of the Economics of Education. 2006;1(2006):307–458. doi: 10.1016/S1574-0692(06)01007-5.
